# Mortality Prediction Using SOFA Score in Critically Ill Surgical and Non-Surgical Patients: Which Parameter Is the Most Valuable?

**DOI:** 10.3390/medicina56060273

**Published:** 2020-06-04

**Authors:** Piotr A. Fuchs, Iwona J. Czech, Łukasz J. Krzych

**Affiliations:** Department of Anaesthesiology and Intensive Care, School of Medicine in Katowice, Medical University of Silesia, 14 Medyków Street, 40752 Katowice, Poland; iwonaczech232@gmail.com (I.J.C.); lkrzych@sum.edu.pl (Ł.J.K.)

**Keywords:** SOFA, mortality, intensive care unit

## Abstract

*Background and Objectives:* assessment systems, such as the Sequential Organ Failure Assessment (SOFA) scale, are routinely used in intensive care units (ICUs) worldwide in order to predict patients’ outcome. We aimed to investigate SOFA’s usefulness in the prognostication of ICU mortality, including an analysis of the importance of its variables. *Materials and Method:* this single-centre observational study covered 905 patients that were admitted from 01.01.2015 to 31.12.2017 to a tertiary mixed ICU. The SOFA score was calculated on ICU admission. The worst results recorded within 24 h post admission were included into the calculation. The assessment was performed within subgroups of surgical (SP) and non-surgical patients (NSP). The subjects were followed-up until ICU discharge or death. ICU mortality was considered to be the outcome. *Results:* ICU mortality reached 35.4% (i.e., 320 deceased out of 905 ICU stays) and it was significantly lower in SP (*n* = 158, 25.3%) as compared with NSP (*n* = 162, 57.9%) (*p* < 0.001). A one-point increase in the SOFA score resulted in 1.35 times higher risk of death in the ICU in the whole studied population. Among the individual variables of SOFA, creatinine concentration was the most powerful in prognostication (OR = 1.92) in univariate analysis, while the Glasgow Coma Scale (GCS) score appeared to be the most important variable in multivariate analysis (OR = 1.8). Mortality prediction using consecutive SOFA variables differed between SP and NSP, as well as between men and women. *Conclusions:* The overall SOFA score predicts mortality to a similar extent in both surgical and non-surgical subjects. However, there are significant differences in prognostication using its particular components.

## 1. Introduction

Simple clinical assessment systems for outcome prediction are gaining in popularity in intensive care units (ICUs) worldwide. In Poland, their routine application on ICU admission is advised by the National Consultant in Anaesthesiology and Intensive Care, with this remaining consistent with the regulations of Polish Ministry of Health [1]. The early identification of subjects who are at risk of poor outcome allows for intensivists to implement personalized treatment or discuss the role of its futility.

The Sequential Organ Failure Assessment (SOFA) scale was primarily designed for mortality prediction in septic patients. It is based on several parameters reflecting multi-organ failure by measuring: concentration of bilirubin; concentration of creatinine; platelet count; PaO_2_/FiO_2_ ratio; Glasgow Coma Scale (GCS) score; and, mean arterial pressure (MAP) value (± the requirement for catecholamines) [2]. The total score ranges from 0 to 24 points, as each parameter is scored from 0 (physiological function) to 4 (worst values) at designated intervals.

In recent years, its use has been extended to other critically ill subjects that were treated in the ICU setting, with acceptable diagnostic accuracy [3,4,5].

In this study, we sought to verify whether the SOFA scale is useful in the prognostication of ICU mortality among surgical and non-surgical patients, with special attention being given to investigating which variables are of the most importance.

## 2. Materials and Methods

**Figure 1:**
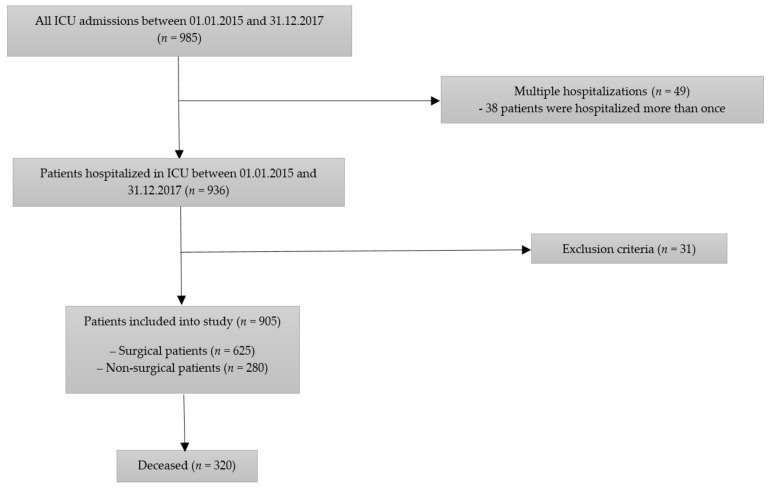
Patients’ flow chart.

In this prospective observational study, a total of 936 consecutive patients admitted to the mixed 10-bed ICU between 1 January 2015 and 31 December 2017 were screened. Among them, 38 persons were hospitalized in the ICU more than once, which gave a total of 985 hospital stays. The exclusion criteria were: age <18 years old, missing data, incorrect national personal identity number or unknown identity, and admissions for organ procurement. A total of 905 patients were enrolled into the study (Figure 1).

The SOFA score was calculated on ICU admission [2]. The data that were necessary to assess the SOFA score were retrieved from medical charts. The worst results recorded within 24 h post admission were included into the calculation. The assessment was performed within subgroups of surgical (SP) and non-surgical patients (NSP). Additional calculations were made according to gender, i.e., for female and male patients. The subjects were followed-up until ICU discharge or death. ICU mortality was considered to be the outcome.

The university Ethics Committee waived the requirement for informed consent due to the anonymous and non-interventional nature of the study, date of approval: 27 March 2018 (KNW/0022/KB/55/18).

A statistical analysis was performed while using StatSoft Statistica version 13.0 software (StatSoft Polska Sp. z.o.o., Kraków, Poland license purchased by the Medical University of Silesia, Katowice, Poland). Quantitative variables are presented as a mean and standard deviation or median and interquartile range (IQR). The qualitative variables are presented as an absolute value and/or percentage. The between-group differences for quantitative variables were verified using a parametric (t-test or ANOVA) or non-parametric tests (U Mann–Whitney or Kruskal–Wallis), with previous verification of their distribution by the Shapiro–Wilk or Smirnov–Kolmogorov test. In the case of qualitative variables, the chi-square test or Fisher’s exact test was used. A receiver-operating characteristic (ROC) curve analysis was used to assess the diagnostic accuracy of SOFA score. A univariate and multivariate logistic regression analysis was conducted in order to assess the possible predictors of ICU mortality. The odds ratios (OR) with 95% confidence intervals were calculated. Each variable is scored from 0 to 4 points, as mentioned before. Univariate and multivariate analyses were conducted while taking points possible to gain for values in each parameter into account. A *p*-value < 0.05 was considered to be significant. 

## 3. Results

The median age of patients was 62 (IQR 50–71) years and there were 493 (54.5%) females in the study group. The baseline SOFA score was 6.33 ± 4.12 points for all patients, 5.51 ± 3.9 points in SP and 8.15 ± 4.02 points in NSP (*p* < 0.001); 5.75 ± 4.03 points in males and 7.01 ± 4.12 points in females (*p* < 0.001). Table 1 presents patients’ characteristics. 

ICU mortality reached 35.4% (i.e., 320 deceased out of 905 ICU stays) and it was significantly lower in SP (*n* = 158, 25.3%) as compared with NSP (*n* = 162, 57.9%) (*p* < 0.001). Mortality was higher in men (*n* = 171, 41.5%) than women (*n* = 149, 30.2%) (*p* < 0.0001).

**Figure 2:**
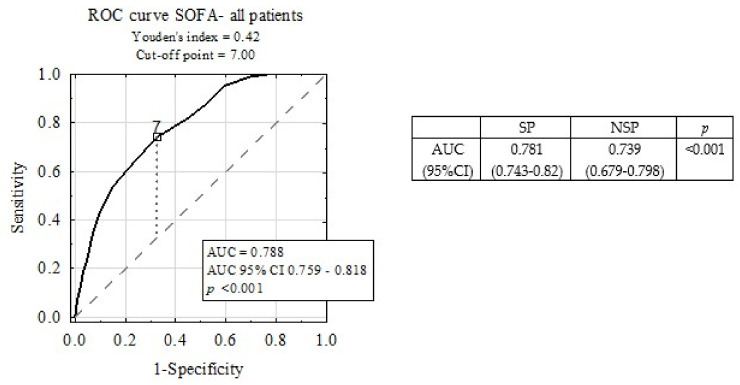
Diagnostic accuracy of Sequential Organ Failure Assessment (SOFA) scale in intensive care unit (ICU) mortality prediction, including sub-group analysis.

Figure 2 depicts the ROC curve for ICU mortality prediction by SOFA scale. The area under the ROC curve (AUC) was 0.788. A subgroup analysis revealed statistically significant differences between AUC for SOFA in the SP and NSP sub-groups (*p* < 0.001).

The SOFA score statistically significantly predicted the outcome in all patients, as well as in the investigated subgroups (Table 2). A one-point increase in the SOFA score resulted in 1.35 times higher risk of death in the ICU in the whole studied population. Table 2 presents detailed odds ratios in different subgroups.

Table 3 shows univariate and multivariate analyses for the prediction of mortality. Among the individual variables of SOFA, creatinine concentration was the most powerful in prognostication in univariate analysis (OR = 1.92). All of the variables were candidates for multivariate analysis. The conducted analyses showed that, individually, each variable had significant impact on mortality; however, after performing multivariate analyses, bilirubin concentration and platelet count didn’t remain significant.

Table 4 and Table 5 present mortality prediction while using each single SOFA variable in the male and female subgroups. Independently, the creatinine concentration was the most powerful variable in both subgroups.

Mortality prediction using consecutive SOFA variables differed between SP and NSP (Table 6 and Table 7). The impact of MAP in NSP patients should be noticed. Bilirubin and creatinine concentrations are only strong predictive factors in SP population.

## 4. Discussion

Our previous study concerning the predictive role of routinely used scoring systems revealed that the SOFA score had the worst ability to predict ICU mortality in comparison with the Simplified Acute Physiology Score (SAPS) II and the Acute Physiology and Chronic Health Evaluation (APACHE) II scale [6]. This finding is of interest, as they are constructed using similar variables. Indeed, this encouraged us to investigate which of SOFA’s elements may distort the total scores and which of its variables are the strongest predictors of the outcome. Moreover, it was important to verify whether prognostication might differ in particular subgroups of critically ill subjects.

In this analysis, we have provided evidence that higher SOFA scores correlate with a compromised outcome, not only in the total population of ICU patients, but also in the subgroups of surgical and non-surgical patients, as well as in men and women with similar odds-ratios. The present study shows that the SOFA scores show good discrimination (AUC 0.788) for predicting the prognosis of the patients hospitalized in the ICU. These observations, together with the simplicity and ease of assessment of SOFA scale, are potential indicators of SOFA’s good diagnostic accuracy in predicting ICU mortality. Our observation is in agreement with that of Shao-Sung et al., whose SOFA score was an independent predictor of long-term mortality in patients with acute myocardial infarction (HR = 1.313; 95% CI 1.191–1.447) [7]. Multivariate analysis of SOFA’s variables revealed that, although the odds-ratios for almost all parameters were lower, the predictive value of the SOFA scale remains strong.

Creatinine concentration was the most powerful variable concerning prognostication in almost all univariate analyses. This is in line with previous studies of critically ill patients [8]. Moreover, multivariate analyses showed that lower GCS scores have been connected with a statistically important increase in mortality. Unfortunately, the impact of sedative agents might distort the results. Surprisingly, bilirubin concentration was only a strong predictive factor in the group of patients after surgeries. Hongyou et al. reported that, in acute myocardial infarction patients, a higher total serum bilirubin level had significantly increased the risk of major adverse cardiac events and cardiovascular mortality, although not the all-cause mortality risk [9]. Surprisingly, although the PaO_2_/FiO_2_ ratio decrease was not associated with a compromised outcome in NSP according to multivariate analysis, the Horowitz index has been reported as a prognostic marker by Wang et al., who indicated that it might be useful for identifying in-hospital mortality of patients with acute pulmonary embolism on admission [10]. In addition, Esteve et al. reported that a simple determination of PaO_2_/FiO_2_ at three hours after ICU admission might be useful in identifying patients at risk immediately after cardiac surgery [11].

Interestingly, our study shows why intensivists should not treat SP and NSP groups uniformly. As these are two completely different populations, different clinical and laboratory parameters may have an impact on the final outcome. The NSP group contains patients after sudden cardiac arrest, or patients with numerous comorbidities who are often hospitalized because of exacerbation of their long-lasting health disorders (i.e., acute-on-chronic disease). Surgical patients in the ICU are hospitalized because of post-surgical complications. Unfortunately, it was impossible to conduct reliable analysis within small subgroups (i.e., depending on co-morbidities), because that would have required larger population and longer time of observation. The top three complications, which contribute to nearly three-quarters of all deaths after non-cardiac surgeries, are myocardial injury after non-cardiac surgery (MINS; 29%), major bleeding (25%), and sepsis (20%), according to the European Society of Cardiology reports [12]; and, pneumonia, myocardial infarction, pulmonary embolism, heart failure, and respiratory failure, according to Larsen [13]. 

What is more, our study reveals some interesting differences in prognostication among men and women. In both subgroups, the GCS score seems to be the most important factor. However, the rest of significant variables differ between men and women. The impact of GCS score might be blurred by the use of sedative agents. Taking that into account, the platelet count is the most important predictor in females and PaO_2_/FiO_2_ ratio has greatest impact on men’s mortality in the ICU. Little is known regarding the impact of gender on the power of SOFA’s elements in the literature. 

Our study has several limitations. First of all, the inherent bias of the single-centre design of the study could not be avoided. Although our study provides information about neurosurgical and gynaecological patients, as well as subjects who have undergone abdominal procedures, we lack data regarding patients who have undergone cardiac, vascular, or orthopaedic surgeries. In addition, we lack detailed information about patients’ comorbidities. What is more, our ICU rarely hospitalizes patients with polytrauma (no trauma emergency department in our location) or out-of-hospital cardiac arrest (no cath-lab in the centre). Another drawback is the rather small study group covering only approx. 1000 subjects, which makes the extrapolation of our results limited. 

We hope that our study can be used by clinicians in interpreting the association between patient’s clinical state and the possibility of death in the ICU, as well as by the authors of next scoring systems in choosing which reliable variables could be used.

## 5. Conclusions

Although the overall SOFA score predicts mortality to a similar extent in both surgical and non-surgical subjects, there are significant differences in prognostication using its particular components. Moreover, the influence of gender might bias this association.

## Figures and Tables

**Table 1:** Characteristics of the study group.

	All Patients (*n* = 905)	SP (*n* = 625)	NSP (*n* = 280)
**Reason for Admission**			
Multi-organ failure	60 (6.63%)	40 (6.4%)	20 (7.14%)
Acute respiratory and circulatory failure	377 (41.66%)	240 (38.4%)	137 (48.93)
Acute respiratory failure	678 (74.92%)	439 (70.24%)	239 (85.3%)
Acute neurological state	202 (22.32%)	160 (25.6%)	42 (15%)
Cardiac arrest	74 (8.18%)	12 (1.92%)	62 (22.14%)
Acute abdomen	66 (7.29%)	59 (9.44%)	7 (2.5%)
Shock	138 (15.25%)	102 (16.32%)	36 (12.86%)
Admission directly from OR	509 (56.24%)	509 (81.44%)	0 (0%)
**Comorbidities**			
Cancer	318 (35.14%)	286 (45.76%)	32 (11.43%)
Cardiac disorders (arrhytmia)	133 (14.7%)	72 (11.52%)	61 (21.79%)
Acute renal faliure	70 (7.73%)	34 (5.44%)	36 (12.85%)
Liver failure	52 (5.75%)	20 (3.2%)	32 (11.43%)
Chronic obstructive pulmonary disease	19 (2.1%)	7 (1.12%)	12 (4.29%)
**Type of surgery**			
Emergency	243 (26.85%)	243 (38.88%)	0
Planned	382 (42.21%%)	382 (61.12%)	0

**Table 2:** Mortality prediction by SOFA score-univariate logistic regression analysis.

Sub-Group	Odds Ratio (95% CI)	*p*
All patients	1.35 (1.29–1.41)	<0.001
Surgical patients	1.34 (1.26–1.43)	<0.001
Non-surgical patients	1.28 (1.18–1.38)	<0.001
Females	1.34 (1.26–1.43)	<0.001
Males	1.35 (1.26–1.44)	<0.001

**Table 3:** Mortality prediction by consecutive SOFA variables–univariate and multivariate logistic regression analysis.

All-Patients	Univariate		Multivariate	
Parameter (Points)	Odds Ratio (95% CI)	*p*	Odds Ratio (95% CI)	*p*
PaO_2_/FiO_2_ ratio	1.67 (1.48–1.88)	<0.001	1.36 (1.17–1.57)	<0.001
Platelet count	1.6 (1.34–1.9)	<0.001	1.23 (0.99–1.52)	0.063
Glasgow Coma Scale	1.82 (1.66–2)	<0.001	1.8 (1.63–1.99)	<0.001
Bilirubin concentration	1.42 (1.22–1.67)	<0.001	1.21 (1–1.18)	0.055
Mean arterial pressure or vasoactive agents required	1.23 (1.14–1.32)	<0.001	1.17 (1.07–1.28)	<0.001
Creatinine concentration	1.92 (1.63–2.27)	<0.001	1.44 (1.18–1.76)	<0.001

**Table 4:** Mortality prediction by SOFA variables in female–univariate and multivariate logistic regression analysis.

Female	Univariate		Multivariate	
Parameter (Points)	Odds Ratio (95% CI)	*p*	Odds Ratio (95% CI)	*p*
PaO_2_/FiO_2_ ratio	1.55 (1.31–1.83)	<0.001	1.21 (0.98–1.48)	0.07
Platelet count	1.8 (1.36–2.39)	<0.001	1.59 (1.25–2.24)	<0.001
Glasgow Coma Scale	1.92 (1.68–2.19)	<0.001	1.87 (1.62–2.16)	<0.001
Bilirubin concentration	1.41 (1.12–1.76)	<0.001	1.23 (0.94–1.6)	0.13
Mean arterial pressure or vasoactive agents required	1.26 (1.14–1.39)	<0.001	1.16 (1.02–1.32)	<0.001
Creatinine concentration	2 (1.56–2.56)	<0.001	1.42 (1.05–1.94)	<0.001

**Table 5:** Mortality prediction by SOFA variables in male–univariate and multivariate logistic regression analysis.

Male	Univariate		Multivariate	
Parameter (Points)	Odds Ratio (95% CI)	*p*	Odds Ratio (95% CI)	*p*
PaO_2_/FiO_2_ ratio	1.75 (1.47–2.09)	<0.001	1.55 (1.25–1.91)	<0.001
Platelet count	1.4 (1.18–1.77)	<0.05	1.01 (0.75–1.34)	0.97
Glasgow Coma Scale	1.7 (1.49–1.93)	<0.001	1.77 (1.53–2.05)	<0.001
Bilirubin concentration	1.38 (1.1–1.73)	<0.05	1.25 (0.94–1.66)	0.12
Mean arterial pressure or vasoactive agents required	1.2 (1.08–1.33)	<0.001	1.21 (1.06–1.38)	<0.05
Creatinine concentration	1.78 (1.42–2.23)	<0.001	1.4 (1.07–1.83)	<0.05

**Table 6:** Mortality prediction by SOFA variables in SP–univariate and multivariate logistic regression analysis.

SP	Univariate		Multivariate	
Parameter (points)	Odds ratio (95% CI)	*p*	Odds ratio (95% CI)	*p*
PaO_2_/FiO_2_ ratio	1.67 (1.42–1.95)	<0.001	1.39 (1.14–1.69)	<0.05
Platelet count	1.39 (1.08–1.8)	<0.05	0.92 (0.67–1.27)	0.61
Glasgow Coma Scale	1.91 (1.69–2.16)	<0.001	1.95 (1.71–2.23)	<0.001
Bilirubin concentration	1.57 (1.28–1.97)	<0.001	1.53 (1.17–1.98)	<0.05
Mean arterial pressure or vasoactive agents required	1.18 (1.08–1.3)	<0.001	1.1 (0.97–1.24)	0.13
Creatinine concentration	2.08 (1.64–2.63)	<0.001	1.58 (1.18–2.12)	<0.05

**Table 7:** Mortality prediction by SOFA variables in NSP–univariate and multivariate logistic regression analysis.

NSP	Univariate		Multivariate	
Parameter (Points)	Odds Ratio (95% CI)	*p*	Odds Ratio (95% CI)	*p*
PaO_2_/FiO_2_ ratio	1.32 (1.07–1.62)	<0.05	1.16 (0.92–1.47)	0.21
Platelet count	1.45 (1.1–1.92)	<0.05	1.42 (1.03–1.95)	<0.05
Glasgow Coma Scale	1.46 (1.26–1.7)	<0.001	1.51 (1.28–1.77)	<0.001
Bilirubin concentration	1.15 (0.9–1.47)	0.26	0.96 (0.72–1.28)	0.77
Mean arterial pressure or vasoactive agents required	1.39 (1.21–1.6)	<0.001	1.39 (1.19–1.62)	<0.001
Creatinine concentration	1.36 (1.07–1.73)	<0.05	1.11 (0.83–1.47)	0.48
